# Editorial: Genome editing in poultry and livestock

**DOI:** 10.3389/fgeed.2026.1946439

**Published:** 2026-08-06

**Authors:** Jiannan Zhang, Lijun Shang

**Affiliations:** 1 Key Laboratory of Bio-Resources and Eco-Environment of Ministry of Education, College of Life Sciences, Sichuan University, Chengdu, China; 2 Animal Disease Prevention and Green Development Key Laboratory of Sichuan Province, College of Life Sciences, Sichuan University, Chengdu, China; 3 School of Human Sciences, London Metropolitan University, London, United Kingdom; 4 Biological Security Center, London Metropolitan University, London, United Kingdom

**Keywords:** animal biotechnology, CRISPR, genetic improvement, genome editing, livestock, poultry

Genome editing is reshaping animal breeding by enabling targeted trait improvement and direct interrogation of gene function in agriculturally important species. In farm animals, its promise has been linked to healthier, more productive, and more sustainable production systems (Tait-Burkard et al., 2018). In poultry and livestock, however, the value of an edit extends beyond molecular efficiency. It also depends on predictable phenotypes, developmental context, welfare, heritability, and responsible use in production systems. The seven contributions to this Research Topic span disease resilience, germ-cell biology, functional genomics, animal bioreactors, precise knock-in, and multiplex editing. Together, they show the field moving from technical advances toward biological validation and responsible applications.

Disease-oriented genome editing is represented by Zanella et al., who challenged *TMPRSS2*-knockout pigs with H1N1pdm09 influenza A virus. The edited pigs developed fewer lung lesions and showed a reduced inflammatory response compared with wild-type animals, while viral shedding and bronchoalveolar lavage viral titers provided complementary information on infection dynamics. This study demonstrates how editing can improve host response to infection and helps separate pathology, tolerance, viral replication, and transmission-related endpoints. It also refines how disease-resilience traits should be conceptualized in breeding: reduced tissue damage, improved clinical tolerance, lower production burden, and transmission control are distinct but complementary objectives. Future work integrating pathology, viral dynamics, immune responses, and contact-transmission models will help define practical disease-control strategies.

Three contributions focus on chickens and highlight the importance of combining editing platforms with developmental and cellular biology. Meng et al. reviewed genetically modified chickens as egg-based bioreactors for therapeutic protein production. Their review emphasizes the scalability of egg production, biologically relevant post-translational processing, and the increasing precision enabled by CRISPR-based engineering. By outlining needs in stable and tissue-specific expression, product quality, egg physiology, biosafety, and regulation, the article defines a clear development path for chicken bioreactor systems. Gu et al. addressed a complementary bottleneck in primordial germ cell (PGC)-mediated genome editing. Exposure of cultured PGCs to 43 °C reduced viability, glycogen content, characteristic gene expression, and gonadal migration, while hyperthermia of recipient embryos also impaired colonization. These findings emphasize that germline editing efficiency depends not only on editing reagents but also on donor-cell metabolic status and recipient-embryo physiology, providing a physiological basis for improving PGC culture, manipulation, and germline transmission.

Kim et al. advanced functional interpretation of non-coding genetic variation in chickens. The authors used CRISPR activation in DF-1 cells to perturb three variant-containing regions associated with nucleotide-related compounds in chicken breast muscle and profiled transcriptomic responses. Activation altered muscle-related pathways, and the strongest region showed alternative promoter activity associated with an atypical *DUSP8* transcript. This work provides a practical route from genome-wide association signals to experimentally testable regulatory hypotheses, which is valuable because many production traits are influenced by non-coding loci whose causal genes remain difficult to resolve. Future extensions using allele-specific perturbation, trait-relevant cell models, and *in vivo* validation will strengthen links among regulatory variants, muscle biology, and breeding value.

The link between editing design and animal bioreactor development was advanced by Wu et al. The authors showed that a reverse-oriented donor increased homology-directed integration of human butyrylcholinesterase at the goat *FGF5* locus relative to the forward design, and they examined orientation effects at the porcine *RAG1* locus. An edited homozygous cell clone was then used for somatic cell nuclear transfer (SCNT), producing a cloned goat with high recombinant protein expression in skin. This study demonstrates that donor architecture can influence knock-in efficiency and transgene expression, an important consideration for large-animal engineering. It also expands livestock bioreactors beyond traditional mammary-gland expression systems. Further work on reproducibility across animals, long-term expression stability, animal health, and biochemical quality of the recovered protein will support progression to reliable biomanufacturing platforms.

Two studies in bovids illustrate the potential of editing production-related traits while emphasizing phenotype, development, and welfare in translational assessment. Singh et al. described an SCNT-derived, biallelic *MSTN*-knockout buffalo calf with a double-muscling phenotype. The report also recorded early postnatal viability, altered serum biochemical indices, and dysregulated inflammatory and chemotactic mediators, providing valuable physiological information beyond the visible muscling phenotype. As a rare proof-of-principle report in buffalo, the study highlights the importance of integrating production-trait evaluation with metabolic monitoring, immune profiling, postnatal development, and welfare assessment in edited large animals. Wang et al. used a single CRISPR/EOCas12i construct to edit *MSTN* and *BLG* in bovine fetal fibroblasts and generated double-edited embryos by SCNT. This work establishes an embryo-stage platform for multiplex editing in cattle and provides a technical basis for simultaneous modification of traits related to muscle and milk-protein composition. Future progression toward agricultural application will benefit from genome-wide specificity assessment, postimplantation developmental evaluation, and phenotypic validation in live animals.

Taken together, these contributions show that genome editing in poultry and livestock is entering a phase in which molecular precision must be matched by phenotypic, developmental, and welfare evaluation. Several priorities emerge. Editing assessment should increasingly include genome-wide sequence changes, structural variants, mosaicism, and unintended donor-vector integration, particularly in knock-in and SCNT-based systems. Functional validation should be aligned with the biological claim, including allele-specific testing for non-coding variants, germline transmission for PGC-based approaches, and longitudinal phenotyping for edited animals. Interpretation of edited-animal phenotypes should consider the combined effects of the intended edit, cell culture, embryo manipulation, and nuclear transfer. Translational evaluation should integrate productivity or product yield with animal welfare, product quality, biosecurity, environmental effects, and regulatory acceptability. The articles in this Research Topic provide technical advances and biological insights across species and applications. They also outline a constructive path for the field: to develop genome editing not only as a precise molecular tool, but as a biologically informed and socially responsible approach to poultry and livestock improvement.

## References

[B1] Tait-Burkard C. Doeschl-Wilson A. McGrew M. J. Archibald A. L. Sang H. M. Houston R. D. (2018). Livestock 2.0 – genome editing for fitter, healthier, and more productive farmed animals. Genome Biol. 19, 204. 10.1186/s13059-018-1583-1 30477539 PMC6258497

